# *T*_1_ Relaxation of Methane in Mixtures with Gaseous Water

**DOI:** 10.1021/acsmeasuresciau.4c00001

**Published:** 2024-03-04

**Authors:** Harm Ridder, Wolfgang Dreher, Jorg Thöming

**Affiliations:** † Chemical Process Engineering (CVT), Faculty of Production Engineering, University of Bremen, Leobener Strasse 6, 28359 Bremen, Germany; ‡ Center for Environmental Research and Sustainable Technology (UFT), Postbox 330 440, 28334 Bremen, Germany; § MAPEX Center for Materials and Processes, University of Bremen, Postbox 330 440, 28334 Bremen, Germany; ∥ Faculty of Chemistry, in Vivo MR Group, University of Bremen, Leobener Str. 7, 28359 Bremen, Germany

**Keywords:** MRI, MRSI, water vapor, T1 relaxation time, chemical shift, operando measurements, high pressure NMR tubes

## Abstract

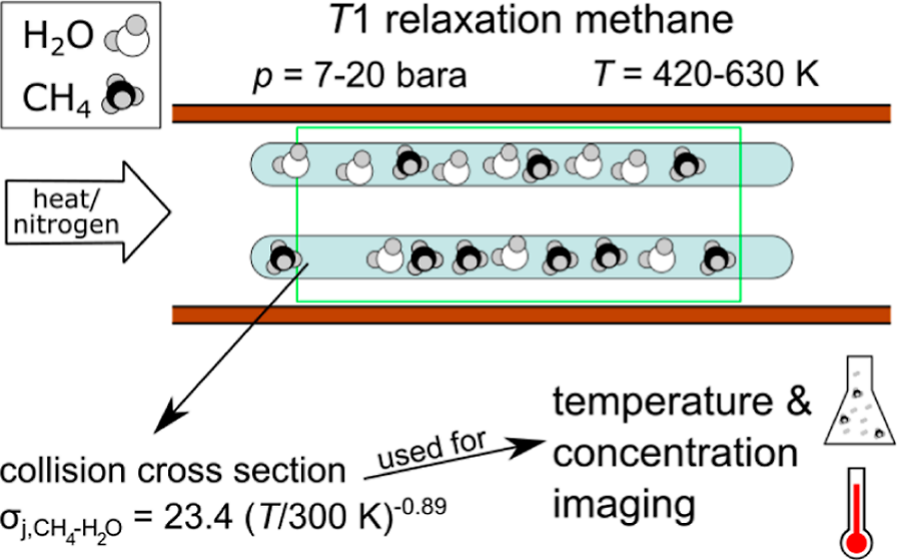

Synthetic, ecofriendly fuels and chemicals can be produced through Power-To-X (PtX) processes. To study such catalytic processes operando and spatially resolved, magnetic resonance imaging (MRI) is a versatile tool. A main issue in the application of MRI in reactive studies is a lack of knowledge about how the gathered signals can be interpreted into reaction data like temperature or species concentration. In this work, the interaction of methane and gaseous water is studied regarding their longitudinal relaxation time *T*_1_ and the chemical shift. To this end, defined quantities of methane-water mixtures were sealed in glass tubes and probed at temperatures between 130 and 360 °C and pressures from 6 to 20 bar. From the obtained *T*_1_ relaxation times, the collision cross section of methane with water σ_*j*,CH_4_-H_2_O_ is derived, which can be used to estimate the temperature and molar concentration of methane during the methanation reaction. The obtained *T*_1_ relaxation times can additionally be used to improve the timing of MRI sequences involving water vapor or methane. Further, details about the measurement workflow and tube preparation are shared.

## 1 Introduction

In chemical engineering, the process intensification of chemical reactions is one of the main tasks aiming to increase the efficiency of production steps.^1^ For some years already, one main focus of scientific developments has been the optimization of processes to store energy chemically in the form of fuels, chemical precursors, or gases in so-called power-to-X (PtX) concepts^2−4^ as a basis for a renewable and circular economy.^5^ PtX processes use the energy-rich hydrogen gas generated from renewable energy sources as an energy feed. The hydrogen is then used to deoxidize a carbon source like CO_2_ or biomass to store a part of the energy therein. Two common products of these reactions are water and methane. Depending on the process, the formation of methane is more or less desirable, while water is practically always an unwanted side product. As a result of this fact and due to the inability of many online measurement systems to measure hydrocarbons and water at the same time, water is usually removed in the post-processing step. However, the presence of water in the catalytically active region is considered a limiting factor both for mass transport and catalytic conversion.^6^

One way to study the presence and interaction of methane and water inside the catalytically active area of a chemical reactor is by using magnetic resonance imaging (MRI) techniques. With MRI, in situ and operando measurements of the reacting zone inside the opaque reactor can be performed. Different MRI techniques offer solutions to spatially map process parameters like velocities,^7,8^ mass transport,^9^ temperature,^10−14^ and species distributions.^15−17^ All techniques have in common the fact that they make use of nuclear magnetic resonance, a phenomenon that can be utilized by applying radio frequency (RF) pulses to a specimen located in a strong magnetic field. The characteristic longitudinal (*T*_1_) and transverse (*T*_2_) relaxation times play an important role in all MRI applications, as they determine how the measured signal evolves over time and how much of the signal is still available after some time. Some of the techniques even base the measurement on the dependencies of these relaxation times, e.g., to estimate the temperature or the local species concentration. In an earlier work,^10^ we introduced such a measurement technique, where a combination of the measured signal and the longitudinal relaxation time *T*_1_ are used to quantify the molar concentration and the temperature of methane during a heterogeneously catalyzed reaction, in particular the methanation reaction. One important factor for this measurement technique is the knowledge of how *T*_1_ surrounds gas molecules. For the methanation reaction, the interactions of methane with CO/CO_2_, H_2_, and H_2_O and a possible inert gas like N_2_ need to be considered. The interaction of methane with other gases is quantified using the effective cross section for the collision of methane with another molecule, *i*, σ_*j*,CH_4_-*i*_. Using this cross section, we can calculate the *T*_1_ relaxation time of methane from theory. The interaction parameters for the collision of methane with CO, CO_2_, and N_2_ have been characterized by Jameson et al.,^20^ while the interaction of methane with H_2_ was investigated in our previous work.^21^ The interaction of methane with (gaseous) water remained an open question, partially because measurements of vapor under defined conditions and without the presence of liquid water are difficult to perform.

In this study, we show the results of measurements of the longitudinal relaxation time *T*_1_ of methane in the presence of water at different temperatures, molar concentrations, and pressures. To enable these measurements, a technique was developed to fill glass tubes with defined amounts of water and methane and seal them. By using a spectroscopic imaging technique for the measurement of *T*_1_, the longitudinal relaxation time of water vapor was also measured. Results for water are given in the CSV file in the Zenodo repository.^22^ This article is based on Chapter 5.2 of the author’s PhD thesis.^19^

## 2 Experimental Section

### 2.1 Setup

In this work, we investigated the longitudinal relaxation time (*T*_1_) of methane in mixtures of the two components at different pressures and temperatures. Since the vapor pressure of water is low under ambient conditions, it is required to perform the measurements at temperatures above 100 °C. This is especially useful for PtX reactions, which usually require temperatures > 100 °C. At increased temperatures, however, temperature homogeneity is difficult to ensure inside an MRI scanner as the setup therein needs to be heated to reach the desired temperature and cooled to prevent overheating of the surroundings. Local cool spots could lead to water condensation, and the water/methane ratio would drastically change. To ensure that the conditions of the mixture are reproducible and to facilitate the measurement process in the MRI scanner, a procedure was developed to seal defined amounts of water and methane inside glass containers. The water was purified using the Omniatap 6 UV/UF (stakpure GmbH, Niederahr, Germany) to an electrical resistivity of about 18 MΩ cm; methane was provided by Linde GmbH (purity 3.7, Pullach, Germany). The glass containers were made from tubes with ID 4 mm and OD 6 mm and were sealed using a blow torch. In contrast to the glass containers used in a prior study which could only be filled up to a pressure slightly below ambient conditions, a quasi-unlimited amount of methane could be sealed inside the glass containers as the preparation procedure involved liquid nitrogen to freeze the methane at the bottom of the tube prior to sealing. The procedure is described in detail in the Supporting Information. Using the presented technique, very large amounts of substance might be filled into a glass container. For safety reasons, the maximum permissible surface tension of the material was taken into account. A list of the prepared glass containers can be found in Table 1 (Supporting Information).

**Figure 1:**
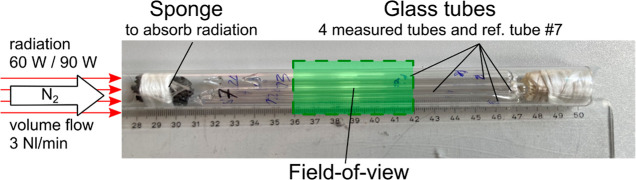
Picture of glass tubes filled with methane and water measured in this study. For better handling, the tubes were placed inside a larger glass tube. The array was positioned inside the heat-resistant MRI reactor described previously^18^ and heated up frontally using the unfocused radiation of a diode laser. Reproduced or adapted with permission from ref (19). Copyright (CC-BY 4.0) 2024 University of Bremen.

**Figure 2:**
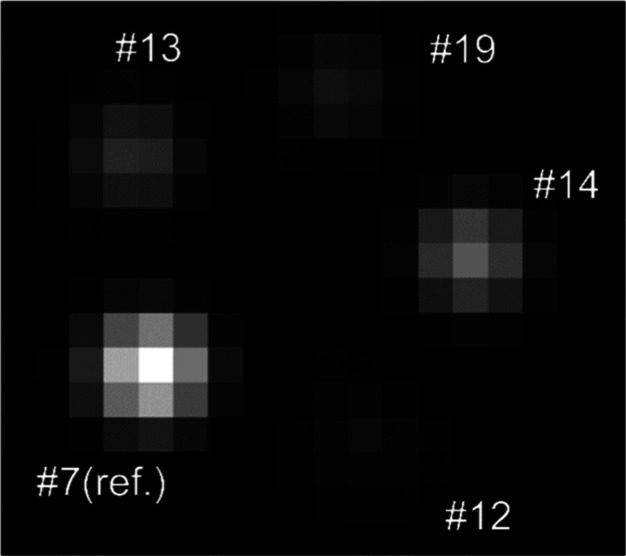
Exemplary transverse MRI image of the methane signal in the central slice presenting the array of five glass tubes of each measurement. Reproduced or adapted with permission from ref (19). Copyright (CC-BY 4.0) 2024 University of Bremen.

The measurements were carried out inside an MRI reactor described in ref (18). For each measurement, there were five glass containers in the reactor (Figures 1 and 2). One of the glass containers was filled with pure methane (no. 7) and was used as a reference for temperature and pressure during all measurements. The remaining glass containers were each filled with varying amounts of methane and water. In total, 19 different glass containers containing water and methane have been measured. The glass containers were frontally heated using a defocused diode laser using a similar setup as presented in our previous work.^21^ A ceramic sponge in front of the glass tubes minimizes reflection of the laser’s radiation due to its irregular structure. To increase the axial heat transport along the glass containers, a constant gas flow of 3 Nl/min nitrogen was applied during all measurements, which transported the heat dissipated inside the sponge to the glass tubes. Each assembly of 4 + 1 glass containers was subjected to two different laser heating levels of 60 and 90 W, respectively. The temperature range in the experiments varied from 130 to 360 °C as a function of the heating level and the observed position of the glass containers. The pressure inside the glass containers ranged from approximately 7 to 20 bar absolute pressure, and the mole fraction of methane ranged from 0.2 to 0.75.

### 2.2 NMR Measurements

A 7 T preclinical NMR imaging system (Biospec 70/20, Bruker Biospin GmbH, Ettlingen, Germany) equipped with a gradient system BGA12S2 (441 mT m^–1^ maximum gradient strength in each direction, 130 μs rise time) was used for all MRI measurements. A circularly polarized volume RF coil (inner diameter of 72 mm; MRI.Tools GmbH, Berlin, Germany) was used for RF excitation and signal detection. The MRI pulse sequence was implemented using the software platform Paravision 5.1.

For the *T*_1_ measurements, we used the same 3D saturation recovery MRSI sequence described in our previous work.^10^ Using two 90° rectangular RF pulses, each followed by spoiler gradients, the magnetization is reduced to a minimum, and then, after a time τ, a third 90° rectangular RF pulse is used to flip the rebuilt magnetization again. Phase encoding gradients are applied in all three spatial directions (*x*, *y*, *z*) immediately after the third pulse, and the emerging free induction decay (FID) is acquired (FOV: 31.5 × 31.5 × 125 mm; matrix size: 21 × 21 × 25 with elliptically reduced *k*-space sampling). From each measurement, the 4D data (1024 × 21 × 21 × 25; *t*, *x*, *y*, *z*) set was zero-filled to a 1024 × 32 × 32 × 25 matrix size and fast Fourier-transformed in the three spatial dimensions. Afterward, it was fitted as a sum of exponentially decaying sinusoids in the time-domain using the matrix pencil method (MPM).^23^ The two signals of methane and water could be separated by their frequency difference of  = 0.56 ppm. As a result of the fitting procedure, a series of amplitudes *S* per voxel (volume element) and per time value τ was acquired for methane and water vapor individually. The data was fitted to the function
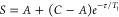
1using the “trust-region-reflective”-algorithm provided by MATLAB (mathworks.com, Version 2017b). The fitting parameters are the maximum signal amplitude *A*, the longitudinal relaxation time *T*_1_, and an error term *C*, which accounts for incomplete saturation of the longitudinal magnetization, e.g., by misadjustment or spatial inhomogeneities of the RF field.

### 2.3 Tube Characterization

**Figure 3:**
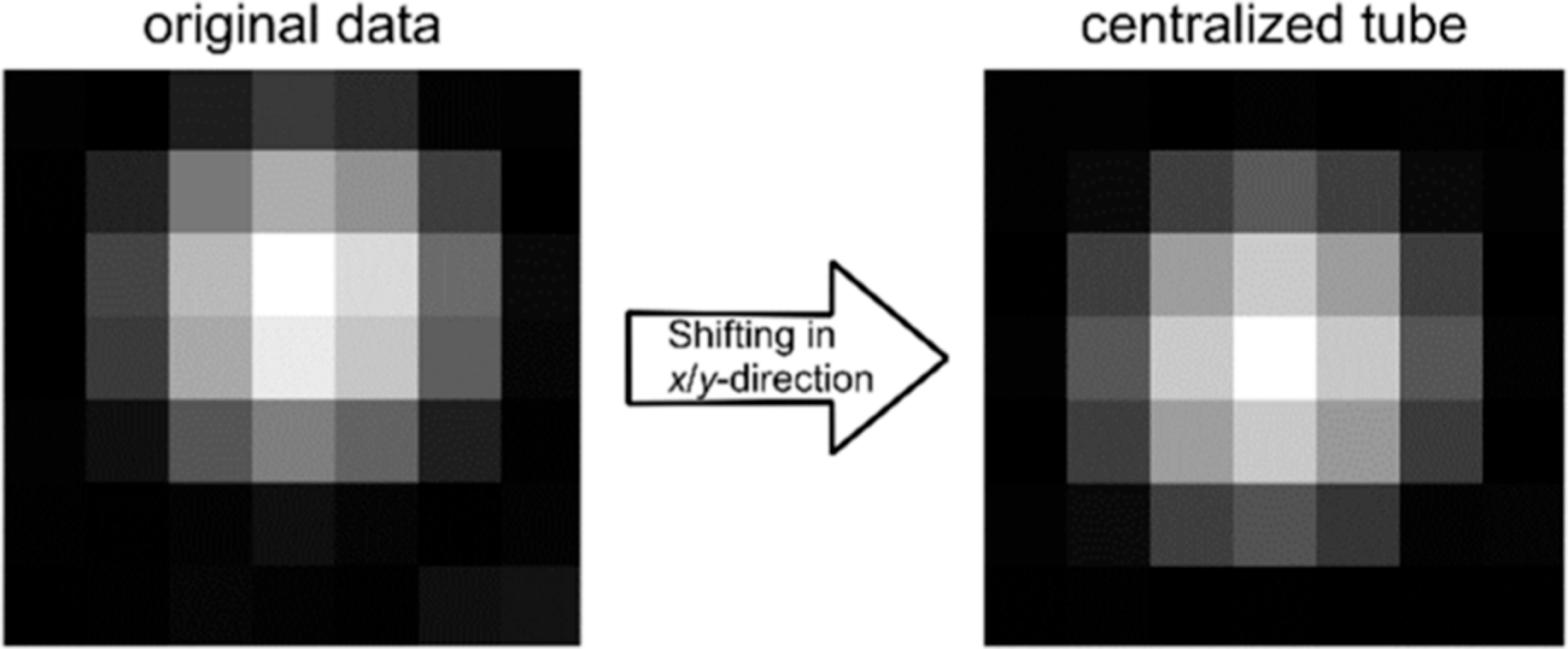
Signal acquired from each tube was slicewise centralized around a single voxel. This way, reliable results from amplitude ratios could be calculated. Reproduced or adapted with permission from ref (19). Copyright (CC-BY 4.0) 2024 University of Bremen.

Using the reference tube and the fitted signal amplitudes in each voxel, temperature *T*, molar concentration ρ, and pressure *p* inside each tube could be quantified. The temperature of the reference tube was calculated using the iterative procedure presented in our previous work,^10^ where the temperature and molar concentration in a volume are calculated from the *T*_1_ relaxation time and the ratio between signal amplitude *A* and the signal amplitude *A*_0_ measured at standard temperature *T*_0_.
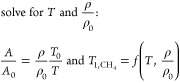
2The mole fraction of methane and water was quantified from the ratio of the signal amplitude of the two signals and averaged over each tube. A factor of 2 was used to account for the different numbers of hydrogen nuclei in the two molecules.
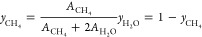
3In the next step, the pressure inside each measured tube was calculated by comparing its signal amplitude to the reference tube’s signal. This factor was applied to the already known pressure inside the reference tube to calculate the pressure inside the measured tube. The pressure inside the reference tube was calculated from the increase in temperature as well as the pressure at standard temperature (index zero) of approximately 9.6 bar absolute pressure, which was determined from *T*_1_ measurements. To improve the quality of the calculated results, either the signal amplitude of methane or of water was used, whichever was larger. Furthermore, only the most central voxel of the tube in each slice was used. Like the methane/water ratio, the pressure was also averaged over each tube.
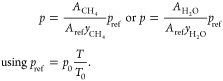
4To calculate reliable results from the amplitude ratios required for temperature and pressure, it was necessary to ensure that the signal in the fitted voxels was distributed symmetrically around a single voxel. Therefore, the MPM fitting procedure was applied to one slice at a time, and the 4D data set was then shifted in *x*- and *y*-dimensions to centralize the signal in that particular slice, as indicated in Figure 3. The procedure was repeated for each of the 13 evaluated slices, and the results were combined into one data set.

In our previous work,^21^ we measured the longitudinal relaxation times *T*_1_ of methane mixed with hydrogen. There, we reported problems with signal inhomogeneities and resulting nonideal signal decays, which led to bad MPM fits. To reduce signal inhomogeneities along the glass tubes, the magnetic field was shimmed with linear and quadratic gradients in the *z*-direction using the signal acquired from the reference tube. Notably, this procedure was repeated before each measurement, as the changes in temperature also changed the local magnetic field. This way, the quality of the MPM fits could be significantly improved.

### 2.4 Determination of σ_*j*,CH_4_-H_2_O_

The main goal of this study is the determination of the cross section for the collision of methane with water, σ_*j*,CH_4_-H_2_O_, which is an essential parameter to calculate the *T*_1_ relaxation of methane in the presence of water. To obtain this parameter, the gathered *T*_1_ data is fitted to the equation proposed by Dong and Bloom^24^
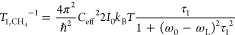
5Here, ℏ = 1.0546 × 10^–34^ J s is the reduced Planck constant, *C*_eff_^2^ the effective spin-rotation constant,^20^*I*_0_ = 5.33 × 10^–47^ kg m^2^ the principal moment of inertia of methane, *k*_B_ = 1.3806 × 10^–23^ J K^–1^ the Boltzmann constant, *T* the temperature, τ_1_ the average time between molecule collisions, ω_0_/2π = 300 MHz the nuclear Larmor frequency, and ω_L_/2π = 16.8 MHz the rotational frequency.

For mixtures, the inverse of the average time between molecule collisions τ_1_^–1^ is calculated as the sum of the molar concentration of species *i*, ρ_*i*_, multiplied by the effective cross section for the collision of methane with species *i*, σ_*j*,CH_4_-i_, and their mean relative gas velocity *v̅*_CH_4_-*i*_^25^
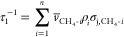
6A least-squares fit is applied to find the value for σ_*j*,CH_4_-H_2_O_, which results in the best agreement of the calculated and measured longitudinal relaxation times *T*_1_.

All calculations were performed by using a MATLAB GUI designed to evaluate MRI data. The functions to evaluate the temperature of the reference tube, centralize the data around a tube, and for fitting σ_*j*,CH_4_-H_2_O_ are added as a special feature for this paper and can all be found in the Zenodo repository alongside the original data that led to these findings. An up to date version of the toolbox can be found on *github.com/HarmRidder/MATLAB-GUI-for-MRI*.

## 3 Results

In this work, the longitudinal relaxation times *T*_1_ of methane in mixtures with gaseous water were measured. From each tube, the 3 × 3 × 13 central voxels were evaluated. However, in some voxels the fit failed, usually because of the influence of the neighboring reference tube, which exhibited a strong signal in comparison to the tubes with lower pressure. Those voxels with failed fits have been ignored.

**Figure 4:**
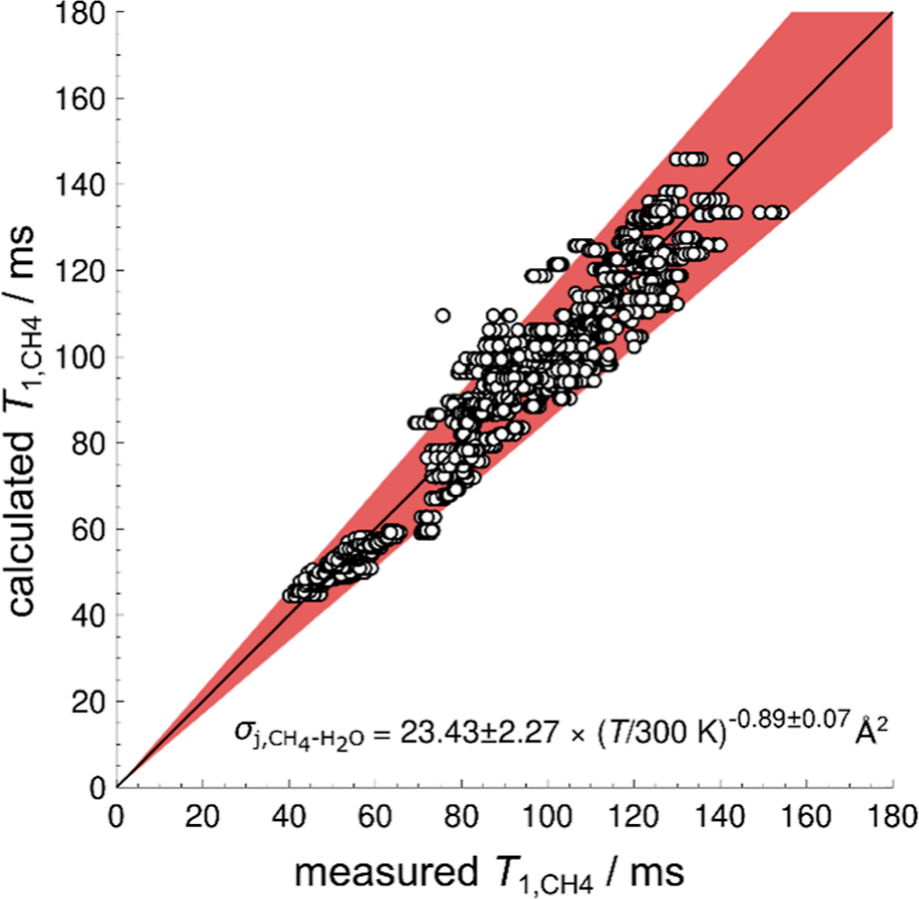
Comparison of measured *T*_1_ relaxation times of methane in a mixture with water and the respective values calculated from the theory of Dong and Bloom (eq 5). The red area indicates a ±15% deviation. The required local temperatures, pressures, and molecule concentrations for the calculation were obtained from the same *T*_1_ measurements by using a reference tube. The interaction parameter σ_*j*,CH_4_-H_2_O_ could be obtained from a least-squares fit as a function of the temperature with a deviation that equals the 95% confidence interval of the mean. Reproduced or adapted with permission from ref (19). Copyright (CC-BY 4.0) 2024 University of Bremen.

From the *T*_1_ relaxation of methane, the effective cross section for the collision of methane with water σ_*j*,CH_4_-H_2_O_ = 23.43 ± 2.27 (*T*/300 K)^−0.89±0.07^ Å^2^ was extracted. Bounds are given as the 95% confidence interval based on the standard error of the mean. Using this, as well as the calculated values for the pressure, temperature, and molar concentration, the *T*_1_ relaxation of methane can be calculated from theory in the presence of water. A comparison between the calculated and measured *T*_1_ times is given in Figure 4. Most data points lie within an accuracy of ±15%, as depicted by the red area.

This study was designed in preparation for spectroscopic magnetic resonance measurements to image heterogeneously catalyzed gas-phase reactions involving methane and water. In the measurements presented in this work, the mean and standard deviation of the difference in frequency between the signals of methane and water was  = 0.56 ± 0.01 ppm, identical to the literature.^26^ From other studies like ref (27), it is known that in the presence of small magnetic field strengths or strong magnetic inhomogeneities, the width in the frequency domain (corresponding to strong signal attenuation in the time domain) prevents signal separation. The mentioned study involves the chemical shift between the methyl (−CH_3_) and methylene (−CH_2_−) groups of hydrocarbons, which exhibit chemical shift difference of approximately 0.4 ppm. Even though the shift between methyl and methylene is slightly smaller in comparison to the shift of 0.56 between water vapor and methane, we experienced the same issue during the measurement of the methanation reaction in one of our previous works,^18^ where the water and methane signal appeared as one peak.

In this work, we were able to keep the line broadening comparably low and, thus, allow proper signal separation. The setup of this work is already advantageous in that simple glass tubes are used instead of a coated honeycomb, thus reducing magnetic field gradients. On top of that, a significant improvement in field homogeneity and subsequently the signal’s , could be achieved by shimming the magnetic field prior to each measurement. The temperature distribution inside the sample–which is caused by the frontal heating of the laser and is a further part of every chemical reactor–is the main factor shifting the local magnetic field strength, subsequently shifting methane’s frequency. This behavior probably arises from the temperature dependence of the magnetic susceptibility of the surrounding materials, like glass or ceramics. Using the scanner’s *z*-gradient coil and the *z*^2^ shim coil, the frequency drift over the probe was minimized. As the methane-filled tube is a mainly one-dimensional object, only the shimming in the *z*-direction was considered. We note in passing that higher order shim coils (e.g., *z*^3^ and *z*^4^) could have improved the magnetic field further but were unavailable for our system.

## 4 Conclusions

In this work, longitudinal relaxation times were determined in mixtures of methane and water vapor inside glass tubes at various temperatures, molar concentrations, and pressures. The results of the measurements were used to quantify the effect of water vapor on the T1 relaxation of methane, as depicted by the effective cross section for the collision of methane with gaseous water σ_*j*,CH_4_-H_2_O_. Further, the frequency shift between water and methane could be quantified to 0.56 ppm with a very narrow window of deviation.

Aside from quantifying the interaction between methane and water vapor, this study yielded other successes. A procedure was developed to fill glass tubes with large amounts of substance, which can then be studied under defined and repeatable conditions as well as high temperatures and pressures. By using a reference glass tube with known pressure, we were able to calculate the temperature and pressure of each glass tube solely based on the information obtained from the MRI measurement. Furthermore, the obtained signal quality could be significantly increased by shimming at increased temperatures.

While the results are promising for future studies and might enable MRI of water and methane during chemical reactions, the possibility to spectroscopically separate methane and water under more challenging conditions is still an open question. Future studies should address the problem of broad line widths, which may hinder proper signal acquisition in some applications. By raising awareness about the challenges of MRI, suitable designs of setups can be found which allow to study reactive processes in-depth and operando.

## Data Availability

The raw data and MATLAB GUI and scripts that led to the findings of this work are made available in a Zenodo repository.^22^ The MATLAB GUI will be kept updated on *github.com/HarmRidder/MATLAB-GUI-for-MRI*. Further information can be given upon request.

## Supporting Information Available

Detailed description of the glass tube preparation procedure; and calculated methane/water ratios used to determine the filling of each tube (PDF)

## Abbreviations

FID: free induction decay
MRI: magnetic resonance imaging
MRSI: magnetic resonance spectroscopic imaging
NMR: nuclear magnetic resonance
PtX: power-to-X
RF: radio frequency
