# Supplementary material for: T1 Relaxation of Methane in Mixtures with Gaseous Water
Source: ACS Meas Sci Au. 2024 Mar 4;4(3):277–82. doi: 10.1021/acsmeasuresciau.4c00001 (PMC11191723; doi:10.1021/acsmeasuresciau.4c00001)
Supplement: Supplementary file 1 — tg4c00001_si_001.pdf [file tg4c00001_si_001.pdf]

Supporting Information of: T_1 relaxation of methane in mixtures with gaseous water

Harm Ridder[a,b], Wolfgang Dreher[d] , Jorg Thöming[a,b,c]

a University of Bremen, Chemical Process Engineering (CVT), Faculty of Production Engineering, Leobener Strasse 6, 28359 Bremen, Germany

b Center for Environmental Research and Sustainable Technology (UFT), Postbox 330 440, 28334 Bremen, Germany

c University of Bremen, MAPEX Center for Materials and Processes, Postbox 330 440, 28334 Bremen, Germany

d University of Bremen, in vivo MR group, Faculty of Chemistry, Leobener Str. NW2,28359 Bremen, Germany

Keywords: MRI, MRSI, water vapor, T1 relaxation time, chemical shift, PtX, operando measurements, high pressure NMR tubes

Appendix A - Description of the glass tube preparation procedure

Creating defined mixtures of water vapor and methane inside a measurement setup is no easy task, as it requires temperatures of at least 100 °C and the amount of water required to match the number of molecules of methane that are typically available in the gas phase is very low. In an open system, where — especially inside an NMR scanner — temperature gradient along the measurement setup are inevitable, it is very difficult to ensure that the exact local molecule ratio of methane and vapor is known.

As a solution to this, a procedure was developed to fixate a known amount of water and methane inside a sealed glass tube. The procedure utilizes the fact that water is liquid at room temperature while methane is gaseous to be able to bring the two substances into the tube one after another. The filling procedure consisted of the following steps (Fig. 6):

1. fill water into the bottom of the glass tube
2. freeze water inside the glass tube by bringing the tube into a bath of undercooled water
3. connect the glass tube to the vacuum pump and gas supply while completely covered inside the bath of undercooled water
4. iteratively draw vacuum and then replace the missing gas with methane to remove the air inside the glass tube
5. set a defined pressure of methane in the glass tube
6. seal the valve at the glass tube and then remove the bath of undercooled water
7. dry the glass tube using a towel
8. put the lower third of the glass tube in a bath of liquid nitrogen to solidify the methane inside the glass tube

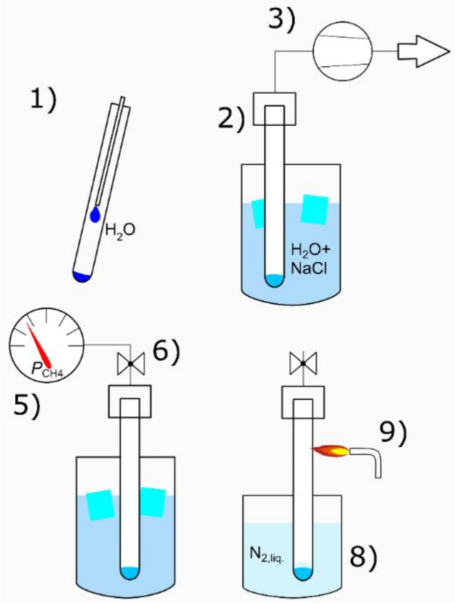

Fig. 1: Scheme of the different steps involved in sealing methane and water inside glass tubes.

9. seal the glass tube using a blow torch

Before the start of the filling procedure, one end of the tube was already sealed. First, the water is filled into the tube using a 10 µl syringe which is typically used for gas chromatographic applications extended by a small tube to be able to reach the bottom of the tube (Step 1). Afterwards the glass tube is almost completely sunk in a bath of undercooled water (-7°C to -2 °C, using NaCl) to freeze the water inside the glass tube (Step 2). The tube is then connected to a small tube array connecting the glass tube to a vacuum pump and a methane supply (Step 3). The connections to the pump and the gas supply are

opened sequentially to draw vacuum and then resupply the removed gas with methane. This way, the remaining air inside the tube could be reduced to a marginal amount, even though the vacuum pump did only reach down to a pressure of 80 mbar (Step 4). In the next step, the pressure regulator of methane was used to set a defined methane pressure inside the glass tube (Step 5). The pressure was checked using two different pressure sensors, one for pressure below ambience and one for pressure above ambience. The resulting pressure is necessary for the calculation of the amount of methane inside the tube. When the desired pressure was reached, the valve near the glass tube was sealed and the bath of undercooled water was removed (Step 6). After drying the tube (Step 7), its lower part was emerged in a bath of liquid nitrogen (Step 8). As a result, solid methane was formed at the bottom of the tube. This ensured that the pressure inside the glass tube was below ambient pressure which allows to seal the tube using a blow torch even when a higher pressure was applied before (Step 9). Though different approaches to sealing water and methane inside a glass tube are theoretically possible, the presented procedure has the benefit that the ratio of water and methane inside the tube is not dependent on the final length of the glass tube which was a quite hard to control parameter as the sealing process was manual.

The amount of water inside the glass tube was calculated as

$$n_{\mathrm{H_2O}} = \frac{V_{\mathrm{H_2O,liq.}}}{V_{\mathrm{m,H_2O,STP}}} \qquad (1)$$

$$\text{with} \quad V_{\mathrm{m,H_2O,STP}} = 1.807 * 10^{-5}\frac{\mathrm{m}^3}{\mathrm{mol}} ,$$

with the volume of water in liquid form $V_{\mathrm{H_2O,liq.}}$ and the molar volume of water at standard temperature and pressure $V_{\mathrm{m,H_2O,STP}}$ provided by the thermo package for python (1). The amount of methane was calculated from the ideal gas law

$$n_{\mathrm{CH_4}} = \frac{V p_{\mathrm{CH_4}}}{RT}, \qquad (2)$$

where V denotes the volume of the glass tube plus the volume of the tubes before the valve, p_{CH_4} is the measured pressure of methane inside the glass tube, $R = 8.314$ J mol^{-1} K^{-1} is the gas constant and T the temperature of the under cooled water. The final pressure dependent on the temperature was calculated using the flasher procedure from the thermo python package (1).

A difficulty of this procedure was to get the exact information on how much water was brought into the glass tube. For the used combinations of water and methane only 0.6 to 13 µl of liquid water was required. The smallest available item to inject the water into the tube was a 10 µl syringe with a scale of 0.2 µl. As a result, amounts of water below 1 µl could only be extracted with reduced accuracy. Further, the amount of water is less than is required to form a drop of water. As a result of the surface tension of the water it could not be ensured that all of the water that was squeezed out of the syringe really stuck to the glass tube's wall. As in this application the exact ratio between methane and water was determined from MRI measurements, the uncertain amount of water did not change the results of this study.

Table 1: Overview over the amounts of water and methane inside the prepared and measured glass containers.

#	$V_{\mathrm{H_2O,liq.}}$ [µL]	$p_{\mathrm{CH_4}}$ [bar] (absolute)	$y_{\mathrm{CH_4}}$ (calc.)	$y_{\mathrm{CH_4}}$ (meas.)
ref	0	10.2	1	-
1	10.1	2.41	0.256	0.22
2	3	6.97	0.755	0.697
3	4.8	3.55	0.5	0.551
4	6.1	5.6	0.503	0.456
5	5.5	1.4	0.252	0.205
6	1.8	4.24	0.745	0.613
7	3.6	3.05	0.495	0.4
8	5.5	0.2	0.042	0
9	9.8	0.2	0.024	0
10	7.4	2.14	0.251	0.241
11	13	0.19	0.019	0
12	2.4	5.43	0.75	0.759

1. Bell C. Thermo Python Package [Internet]. Cambridge, USA: Massachusetts Institute of Technology; 2021. DOI: 10.5281/zenodo.4892196
